# ATP-citrate lyase inhibitor improves ectopic lipid accumulation in the kidney in a db/db mouse model

**DOI:** 10.3389/fendo.2022.914865

**Published:** 2022-12-08

**Authors:** Zishun Zhan, Aimei Li, Wei Zhang, Xueqin Wu, Jinrong He, Zhi Li, Yanchun Li, Jian Sun, Hao Zhang

**Affiliations:** ^1^ Department of Nephrology, The Third Xiangya Hospital, Central South University, Changsha, Hunan, China; ^2^ The Critical Kidney Disease Research Center of Central South University, Changsha, Hunan, China; ^3^ Division of Biological Sciences, Department of Medicine, University of Chicago, Chicago, Chicago, IL, United States; ^4^ Department of Rheumatology and Immunology, The Third Xiangya Hospital, Central South University, Changsha, Hunan, China

**Keywords:** BMS-303141, ACL, ATP-citrate lyase, ectopic lipid accumulation, renal inflammation, obesity-related nephropathy

## Abstract

**Aim:**

We evaluated a novel treatment for obesity-related renal, an ATP-citrate lyase (ACL) inhibitor, to attenuate ectopic lipid accumulation (ELA) in the kidney and the ensuing inflammation.

**Materials and methods:**

An ACL inhibitor was administered intragastrically to 12-week-old db/db mice for 30 days. The appearance of ELA was observed by staining kidney sections with Oil Red O, and the differences in tissue lipid metabolites were assessed by mass spectrometry. The anti-obesity and renoprotection effects of ACL inhibitors were observed by histological examination and multiple biochemical assays.

**Results:**

Using the AutoDock Vina application, we determined that among the four known ACL inhibitors (SB-204990, ETC-1002, NDI-091143, and BMS-303141), BMS-303141 had the highest affinity for ACL and reduced ACL expression in the kidneys of db/db mice. We reported that BMS-303141 administration could decrease the levels of serum lipid and renal lipogenic enzymes acetyl-CoA carboxylase (ACC), fatty acid synthase (FAS), HMG-CoA reductase (HMGCR), and diminish renal ELA in db/db mice. In addition, we found that reducing ELA improved renal injuries, inflammation, and tubulointerstitial fibrosis.

**Conclusion:**

ACL inhibitor BMS-303141 protects against obesity-related renal injuries.

## Introduction

Obesity, or excessive adipose tissue, is a worldwide epidemic. The World Health Organization report affirms that obesity and its complications pose a medical and economic burden that is a global concern (1). Diabetes mellitus, the major cause of chronic kidney disease (CKD) (2, 3), is becoming increasingly common, primarily owing to increases in the prevalence of obesity (4).

Renal ectopic lipid accumulation (ELA) describes the excess of free fatty acids from adipose tissue accumulating in the kidneys to then esterify to triglycerides as ectopic fat deposition (5). In obesity-related renal injuries, ELA and inflammation are both important pathological processes. ELA is deemed to be a critical cause oflipotoxicity in the kidneys (3, 6), which enhances mitochondrial dysfunction, oxidative stress, and endoplasmic reticulum stress, and finally stimulates pro-inflammatory and profibrogenic pathways (7). Excess lipolysis, which is influenced by tumor necrosis factor-alpha (TNF-α) during chronic tissue inflammation, may result in re-esterification and ELA, and impaired insulin signaling (8, 9). In obesity-related nephropathy, the process of ELA is quite important.

ATP-citrate lyase (ACL) catalyzes the conversion of citrate to acetyl CoA, which is a substrate for the biosynthesis of fatty acids and cholesterol. In the non-alcoholic fatty liver disease mouse model, ACL is highly induced in the liver and intensifies ELA (10–12). We have previously reported that acetyl-CoA not only participates in the ELA process but is also a source of histone acetylation, which results in the upregulation of the epigenetic process of ELA and profibrotic genes in mesangial cells (13, 14). Nonetheless, the underlying beneficial effect of ACL inhibition on obesity-related renal injuries *in vivo* has not been investigated.

Currently, there are four known ACL inhibitors, ETC-1002, SB-204990, NDI-091143, and BMS-303141. ETC-1002 (bempedoic acid) is a new once-daily oral hypolipidemic agent that has proven to be efficient in reducing low-density lipoprotein cholesterol (LDL-C). SB-204990 exerts tumor-suppressive effects by inhibiting ACL in HepG2 cells attenuating aerobic glycolysis in malignant cells, reducing tumor growth, and inducing differentiation (15, 16). NDI-091143, a novel macrocyclic compound 2, has been identified as a potent ACL inhibitor for conformational restriction, which shows potent ACL inhibitory activity and binding affinity comparable to the positive control (8). BMS-303141 also plays a similar role in lipid-lowering as the above chemicals, and apart from that, there is cell apoptosis induction in hepatocellular carcinoma *via* the p-eIF2α/ATF4/CHOP axis (9, 17). However, there are no detailed reports on treating obesity-related nephropathy in mouse models with these ACL inhibitors.

According to previous studies, modifying ACL function could be a promising therapeutic target *in vivo*. We aimed to evaluate the role of an ATP-citrate lyase inhibitor as a novel treatment for obesity-related nephropathy by attenuating ELA in the kidney and the ensuing inflammation.

## Research design and methods

### Molecular docking

From the four known chemicals, ETC-1002, SB-204990, NDI-091143, and BMS-303141, we screened the ACL inhibitor with the highest affinity. First, the 3-D structural diagrams of the molecules were downloaded from the PubChem database (https://pubchem.ncbi.nlm.nih.gov/). The Open Babel software (http://openbabel.org/wiki/Main_Page) was then used to transform the chemical file format from SDF to Mol2 format (18). The mouse protein structure was obtained from UniProt (https://www.uniprot.org/), which was then used to add hydrogen, compute charges, and show rotatable keys before format transforming. Each molecule was then set as the ligand, and the corresponding target proteins were utilized as molecular docking receptors. The findings were analyzed using AutoDock Vina and Discovery Studio 3.0, and the PyMOL software was used to interpret them.

### Animal studies

We purchased 8-week-old male C57BLKS/J mice from GemPharmatech Co., Ltd. (Nanjing, China). Adult C57BLKS/J db/db mice were severely obese, had type-2 diabetes mellitus (T2DM), and were highly prone to renal injuries, which met the criteria (19, 20). Thereafter, we categorized male C57BLKS/J db/m and db/db mice into four groups, each of them containing eight mice, fed with a regular chow diet. Bodyweight and glycemic levels were recorded every two weeks using a Contour glucometer (Bayer, Leverkusen, Germany). BMS-303141 (Selleckchem, Munich, Germany) was administered orally as a suspension in 0.5% sodium carboxymethyl cellulose solution (Selleckchem, Munich, Germany) once daily at 50 mg/kg (intragastric) for 30 days (9); BMS-303141 was administered to db/db mice (n = 8) and age and sex-paired db/m mice (n = 8) starting at the age of 12 weeks. Control db/db mice (n = 8) and control db/m mice (n = 8) were administered sterile water for injection once a day for 30 days.

After 30 days, the mice were euthanized and their kidneys were excised. Both the renal cortex and medulla were utilized to conduct experiments such as western blotting. The excised kidneys underwent rapid dissection before being introduced into buffered formalin (10%) for consecutive biochemical and histologic analyses. Blood samples obtained from the left ventricle and the plasma for the subsequent analyses were stored at −80°C. The Central South University’s Institutional Ethical Committee approved all animal protocols for our research.

### Blood and urine parameters measurements

All blood specimens were obtained after the mice were subjected to overnight fasting. Urinary creatinine, plasma cholesterol, serum blood urea nitrogen, plasma triglyceride, and serum creatinine were evaluated individually by an autoanalyzer (BS-430, MindRay Co. Ltd., Shenzhen, China).

### Histology and immunohistochemistry

A 10% buffered formalin solution was utilized to fix the kidney specimen samples after harvesting. The samples were then immersed in a solution containing paraffin and underwent a triple processing staining using the following: hematoxylin-eosin (H&E) staining, Masson’s trichrome staining, and periodic acid-Schiff (PAS) staining. The PAS-stained slides were utilized for a 5-grade approach to semiquantify the glomerulosclerosis index (GSI) as the glomerulosclerosis scores (21). More than 30 glomeruli samples that were incised *via* the vascular pole were tallied for each kidney, and the mean was calculated. Additionally, the frozen sections were sectioned at a thickness of 5 μm to facilitate the Oil Red O staining. Tubular dilatation and epithelial desquamation with interstitial expansions were graded on a scale of 0-4 for renal tubulointerstitial lesions (TIL) (22). In random fields, the regions of fibrotic lesions that were obtained from the cortical interstitium were computed and presented as a proportion of the fibrotic region compared to the total area. Photomicrographs of the regions or sections (n = 3 per animal) at 200x magnification were obtained employing an optical microscope (Olympus, Japan), and the images were evaluated using the Image-Pro Plus program. Immunohistochemical (IHC) staining for a murine eosinophil marker, F4/80 (1:200, Cell Signaling Technology, 70076), and ACL (1:200, ZENBIO, 200359) was performed; the F4/80 staining intensity for semiquantitative evaluation was calibrated as the integrated optical density (22).

### Electronic microscopy

A solution of 2% glutaraldehyde in 0.1 M phosphate buffer (pH 7.4) was used in fixing the kidney specimens, which were subsequently postfixed in 1% osmium tetroxide. The glomerular basement membrane (GBM) was revealed using an electronic microscope, and the thickness was determined using ImageJ, an image processing program (23).

### Western blotting

We extracted the total protein and the nuclear proteins from the kidney renal cortex and cells, and determined their relative concentrations with the aid of ultraviolet spectrophotometry. The proteins on the membrane were displayed with the aid of Image Studio software, whereas band intensities were semiquantitatively evaluated using the ImageJ. The antibodies used (dilutions, catalog number) included ACL (1:2000, Abcam, ab40793), fatty acid synthase (FAS, 1:1000, Abcam, ab128870), acetyl-CoA carboxylase (ACC, 1:2000, Cell Signaling Technology, 3676), HMG-CoA reductase (HMGCR, 1:1000, Abcam, ab174830), transforming growth factor-β1 (TGF-β1, 1:1000, Proteintech, 21898-1-AP), fibronectin (1:1000, Proteintech, 15613-1-AP), β−actin (1:10000, Proteintech, 66009-1-Ig), E-cadherin (1:2000, Proteintech, 20874-1-AP), nephrin (1:2000, Abcam, ab216341), α-smooth muscle actin (α -SMA; 1:1000, ZENBIO, 380653), and collagen I (1:1000, Proteintech, 14695-1-AP).

### Reverse transcription-polymerase chain reaction

An omega kit (Omega Bio-Tek China) was used in the extraction of total RNA. A Thermoscript RT kit (Thermo Fisher Scientific, USA) was utilized in the generation of the first strand of cDNAs, which were amplified by SYBR Green and the Roche LightCycler 480 system. In a 20 μL reaction volume, the first strand of complementary DNA (cDNA) was produced from equal quantities of total RNA (2 μg) utilizing reverse transcriptase and Oligo(dT) primers (Vazyme, China). Real-time quantitative polymerase chain reaction (Q-PCR) evaluations were done with SYBR FAST qPCR Master Mix (Vazyme, China) and primers, as shown in **Table 1**. The threshold cycle, expressed as Ct, is the number of cycles required to produce a fluorescence signal. Ct values are inversely proportional to the initial template number. Fold changes in mRNA expression were computed as per the comparative Ct methodology (2-ΔΔCt).

**Table 1:** Oligonucleotide primers used in the study.

	Sequence, 5’-3’	
Primer	Forward	Reverse
Acly	GTGGAGAAGATTACCACCTCCA	TTCCTAGCACAAAGATGCCATTGA
Acaca	GGCTTACGTCTGGGACAATAA	CGGATCTGCTTCAGGACATAG
Fasn	CGTGTGACCGCCATCTATATC	GATACCACCAGAGACCGTTATG
Hmgcr	GGTGGTGAGAGAGGTGTTAAAG	GCGATGTAGATAGCAGTGACAA
Mcp-1 Tnf-α β-actin	CAAGAAGGAATGGGTCCAGA TCAGCCTCTTCTCATTCCTG TGTTTGAGACCTTCAACACC	TGAGGTGGTTGTGGAAAAGG CAGGCTTGTCACTCGAATTT CGCTCATTGCCGATAGTGAT

### Lipidomic analysis

Mouse renal cortex tissue was extracted from the four groups of mice and all samples were weighed over 100 μg and prepared as previously described by Shanghai BioProfile Technology Co. Ltd.

Lipidomic profiling was conducted using the ultra-performance liquid chromatography-mass spectrometry system (Q Exactive Plus, Thermo Scientific) at Shanghai BioProfile Biotechnology (Shanghai, China). The mass spectrometric data were analyzed using the LipidSearch 4.1.30 software (Thermo Scientific, USA).

### Statistical analysis

The mean ± SD was utilized to present all the data above. After gathering the experiment data, the Shapiro-Wilk normality test was performed if the sample size <8, and if the sample size=8 the D’Agostino and Pearson normality test was performed. Comparisons of Gaussian distribution data groups were performed through a two-way ANOVA with a Tukey posthoc test for differences between the four groups where necessary. The non-Gaussian distribution data were subjected to Kruskal-Wallis one-way ANOVA with Dunn’s multiple comparison test. All analyses were completed with GraphPad Prism version 8.4 Statistical Software (La Jolla, CA, USA), and a P <0.05 was considered statistically significant. Differences between the two groups were ascertained using a Student’s t-test.

## Results

### Docking of inhibitors with ACL to screen for the highest affinity inhibitor

**Figure 1:**
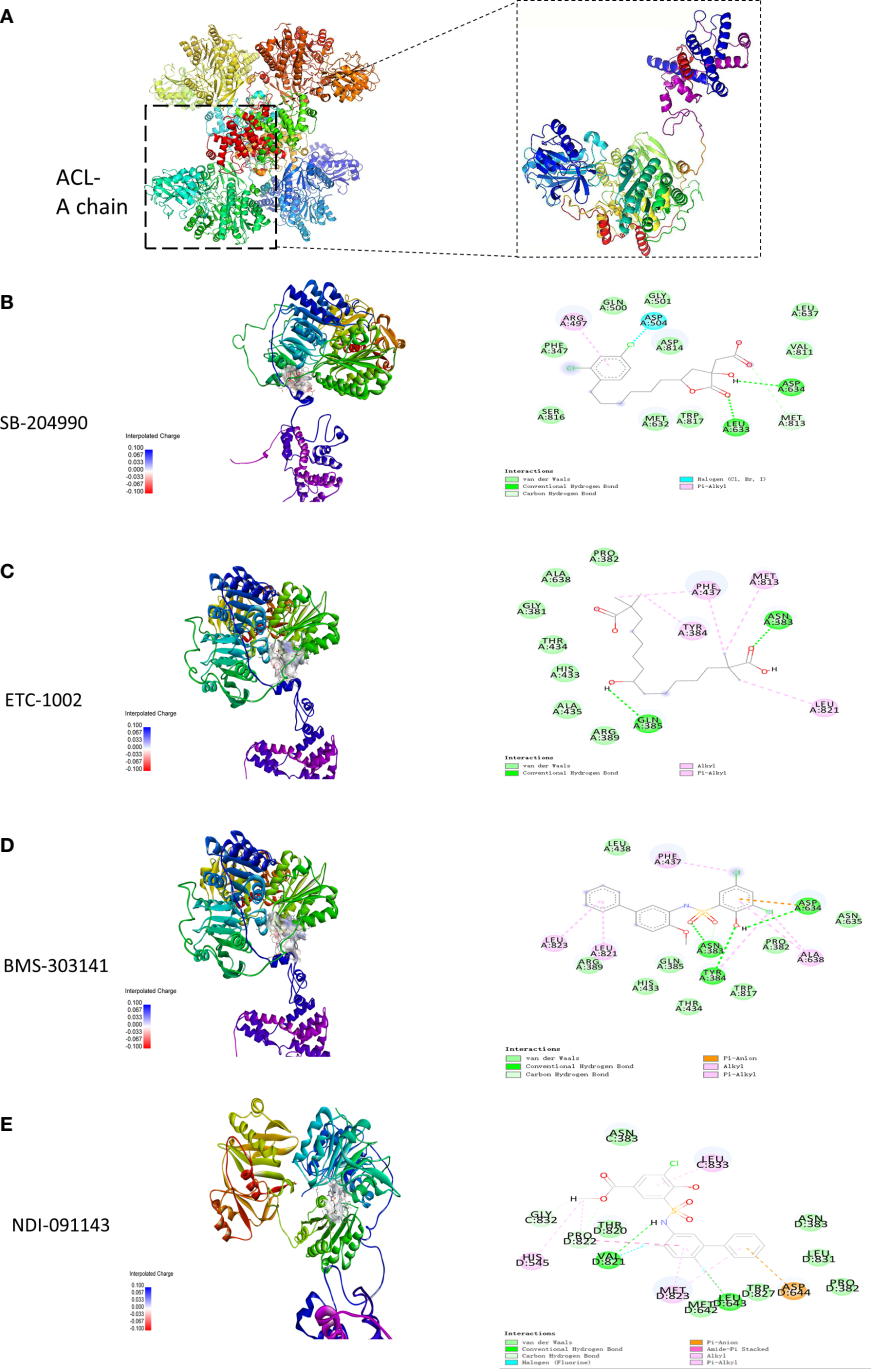
**(A)** Three-dimensional structure of ACL. **(B-E)** Modeled interactions of SB-204990, ETC-1002, BMS-303141, and NDI-091143 with ACL and the docking binding pocket of chemicals with ACL after homology modeling. The chemical in the binding model has been covered with spheres. Amino acid residues of ACL that participated in binding models are shown in green and purple.

The results exported by AutoDock software are shown in **Table 2** and **Figure 1**. The results of the docking complex were processed by Discovery Studio 3.0 and PyMOL software (**Figures 1A–E**). The binding energies of the four docking models formed with the core protein were lower than −5.0 kcal/mol. Our recent docking findings implied that BMS-303141 formed a hydrogen bond with the essential active residues ASP:634, ASN:383, and TYR:384 of ACL (**Figure 1A**) on the A chain. In addition to hydrogen bonding, multiple hydrophobic reactions between BMS-303141 and aromatic residues of ACL were recorded, e.g., LEU: 821 and 823; ALA: 638; PHE: 437 on the A chain (**Figure 1D**). Furthermore, Discovery Studio 3.0 software was used to dock the active molecule and the corresponding target protein, and the LibDock score of the formed docking model was greater than 100. As seen in **Table 2**, among the binding energies for the four docked complex crystal structures, BMS-303141 showed a binding energy of < −8 kcal/mol, and root-mean-square difference (RMSD)<4 Å, indicating that it had the highest affinity for a specific protein.

**Table 2:** Results of AutoDock-Vina docking study.

Receptor(UniProt Code #)	Compound	PubChem CID	Structure	Binding Energy (kcal/mol)	RMSD(Å)
ACL (#Q91V92)	BMS-303141	16747776	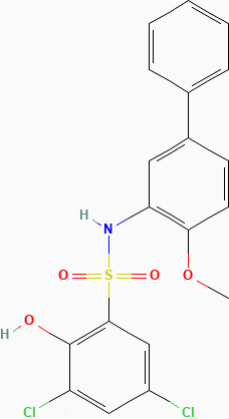	-8.6	2.595
	SB-204990	10340264	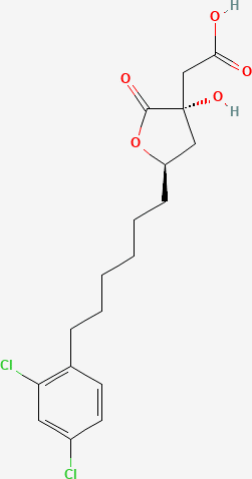	-6.4	1.635
	ETC-1002	10472693	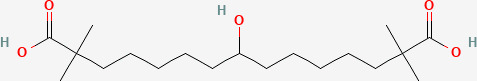	-5.6	1.304
	NDI-091143	137796782	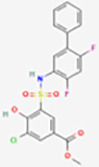	-7.3	1.724

### Effects of BMS-303141 on systemic metabolism and body fat deposition in db/db mice

**Figure 2:**
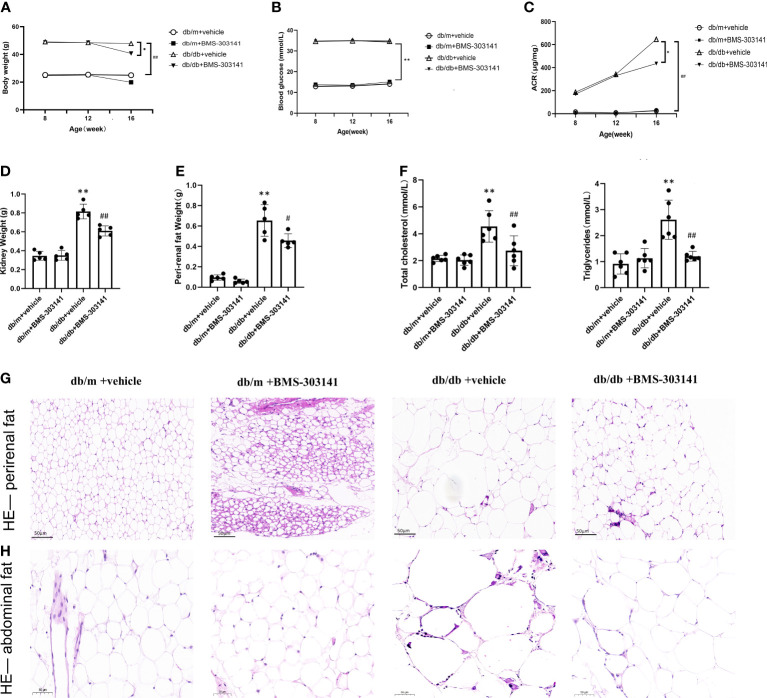
Effect of BMS-303141 on systemic metabolic parameters and body fat deposition in db/m and db/db mice. **(A)** Changes in body weight for 8-week-old db/m and db/db mice, n=8 per each group; **(B)** Differences in fasting blood glucose, n=8 per each group; **(C)** Urinary ACR from db/m and db/db mice for eight weeks, n=8 per each group; **(D, E)** Kidney and perirenal weight, n=5 per each group; **(F)** Changes in plasma lipids of eight weeks, total cholesterol and total triglycerides, n=6 per each group; **(G, H)** H&E staining of perirenal fat and abdominal fat (magnification 200x); Each bar and line represent the mean ± SD of four separate experiments. *P < 0.05, **P < 0.01 versus db/m + vehicle;#P < 0.05, ##P < 0.01 versus db/db + BMS-303141 group.

The db/db mice had higher body weight, visceral fat deposition, and metabolic levels than the db/m controls and eventually developed severe hyperglycemia and hyperlipidemia. In contrast, the BMS-303141-treated db/db mice exhibited reduced body weight and blood fat (**Figures 2A, F**). However, BMS-303141 did not improve the hyperglycemia (**Figure 2B**). The inhibitor alleviated the increase in albuminuria in db/db mice, which indicated that the impaired glomerular filtration function had improved (**Figure 2C**). In addition, the weight change in the visceral fat deposition and the H&E staining of perirenal fat and abdominal fat showed differences in lipid droplet density between the treated and untreated db/db mice, which demonstrated the lipid-lowering effect of the molecule throughout the body (**Figures 2D, E, G, H**).

### BMS-303141 relieved renal ELA and downregulated key lipogenic genes in db/db mice

**Figure 3:**
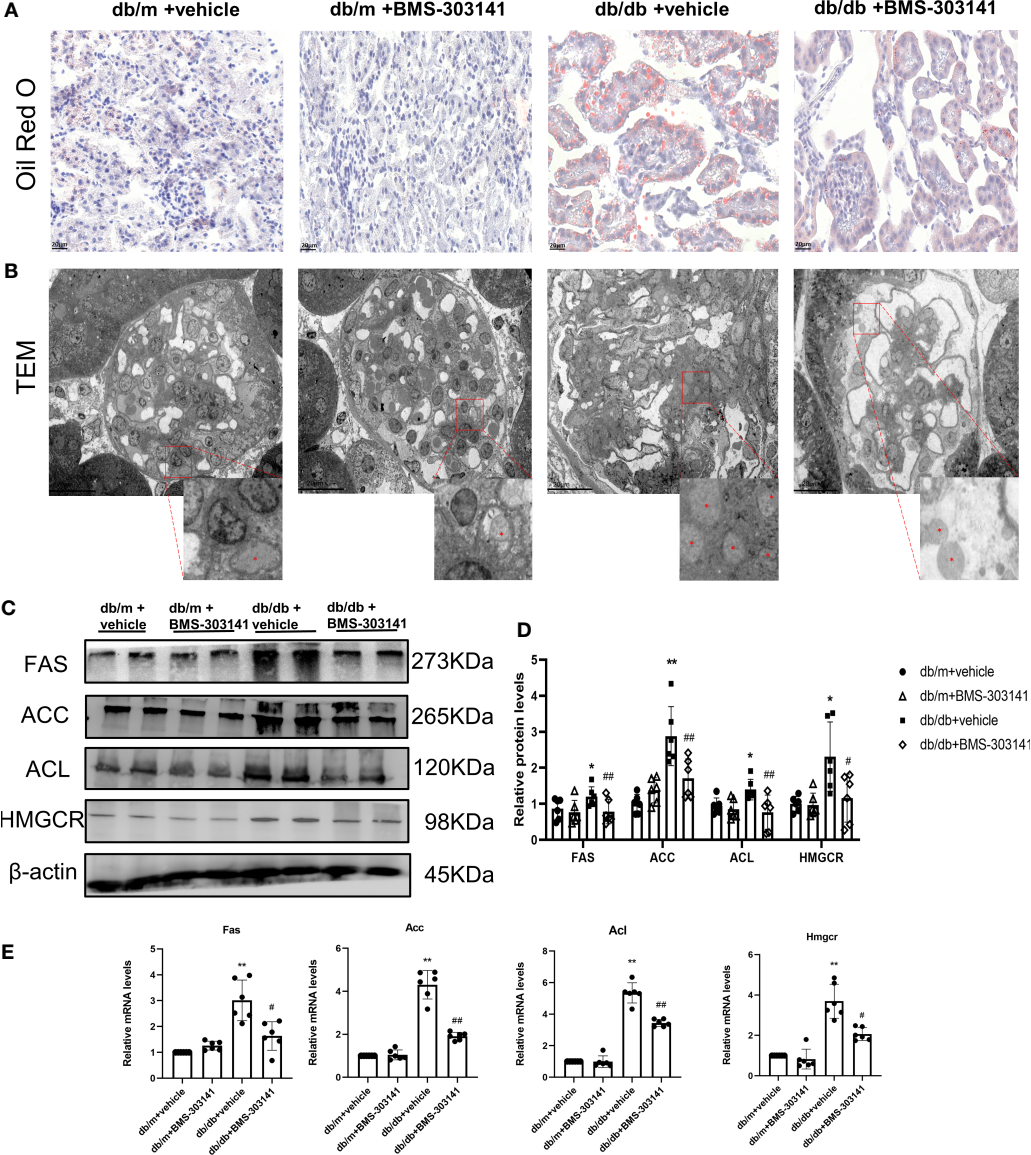
Effects of BMS-303141 on renal ELA and the modulation of critical lipogenic and fibrotic genes. **(A)** Representative kidney cortex sections-related staining Oil Red O (magnification 400x) from db/m and db/db mice for 16 weeks; **(B)** Transmission electron microscopic examination of glomeruli (magnification 500x). The magnified images are the boxed regions, lipid droplets are marked with asterisks (*). C&D) Western blots **(C)** and densitometric semiquantitation **(D)** of specified proteins in the kidney cortex from db/m and db/db mice; *P < 0.05, **P < 0.01 versus db/m + vehicle;#P < 0.05, ##P < 0.01 versus db/db + vehicle group, n = 6 per each group; **(E)** Quantitative real-time PCR analysis of *Acl, Hmgcr, Acc*, and *Fas* transcripts in the kidney cortex of db/m and db/db mice, *P < 0.05, **P < 0.01 versus db/m + vehicle; #P < 0.05, ##P < 0.01 versus db/db + vehicle group, n = 6 per each group. The mean ± SD is represented by each bar in the four independent experiments.

As BMS-303141 decreased droplet density, we further identified its role in lipid metabolism in db/db mice. Oil Red O showed significant lipid deposition in the glomeruli and renal tubular regions between db/m and db/db mice (**Supplementary Figure 1A**), which was decreased remarkably by BMS-303141 (**Figure 3A**). Electron microscopic analysis of the mesangial cells in the kidneys of untreated db/db mice additionally confirmed clear lipid droplet deposition, significantly more than the other three groups of mice (**Figure 3B**). After administering BMS-303141, western blotting results (**Figures 3C, D**) and IHC results (**Supplementary Figure 1C**) showed a reduction in the levels of ACL protein and those proteins associated with several rate-limiting enzymes of lipid synthesis (ACC, FAS, and HMGCR), and Q-PCR analyses showed a decrease in *Hmgcr, Acc*, *Acl*, and *Fas* transcripts in the renal cortex (**Figure 3E**), which suggested BMS-303141 can relieve renal ELA and downregulate key lipogenic genes in db/db mice.

### BMS-303141 suppressed lipid production with altered lipid profiles in db/db mice

**Figure 4:**
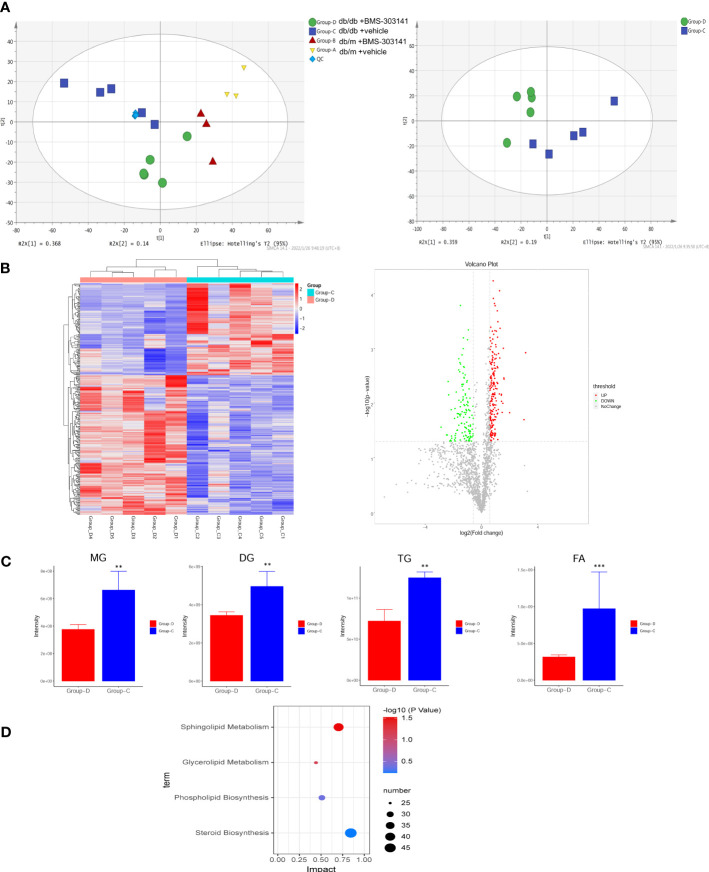
BMS-303141 suppresses lipid production with altered lipid profiles. **(A)** PCA analysis of the original four groups and both db/db mouse groups. **(B)** Heatmap and Volcano plot between two db/db mice groups with fold change. **(C)** The relative contents of some of the different lipid classes detected in the Group-D vs Group-C comparison. Group **(D)** KEGG pathway analysis. The mean ± SD of four different tests is represented by each bar. *P < 0.05, **P < 0.01, ***P < 0.001 Group-D vs Group-C, n = 5. ChE, cholesterol ester; DG, diglyceride; MG, monoglyceride; TG, triglyceride; FA, fatty acid.

Lipidomic analysis of lipid profiles identified 42 lipid classes and 1835 lipid species of lipid metabolites in total. Principal component analysis was used to test differences between the four groups, to eliminate the trend of separation within each group (**Figure 4A**). Our experiments to prove the effectiveness of the inhibitor molecules and to explore the effect of ACL inhibition on the metabolic pathway showed statistically significant lipid species in both treated and untreated db/db groups. We found differences in predominant lipid species, particularly in glyceride and fatty acid, revealing the level of ectopic lipid deposition in the kidney that caused obesity-associated inflammation, resulting in the progression of obesity-related CKD (**Figure 4C**). Whereas only 433 metabolites (**Figure 4B**, **Supplementary Table 1**) were remarkably altered (fold change ≥ 1.5, p < 0.05), KEGG pathway enrichment revealed that the differential lipid metabolites were mainly centered on sphingolipid metabolism and glycerolipid metabolism (**Figure 4D**). Sphingolipids are the main source of biomembrane structure, especially in the cell membrane. The decrease in glycerides may suggest that lipocatabolism could be attenuated in the kidney in a glycometabolism disorder, in addition to insulin resistance.

### BMS-303141 attenuated renal injuries and inflammation in db/db mice

**Figure 5:**
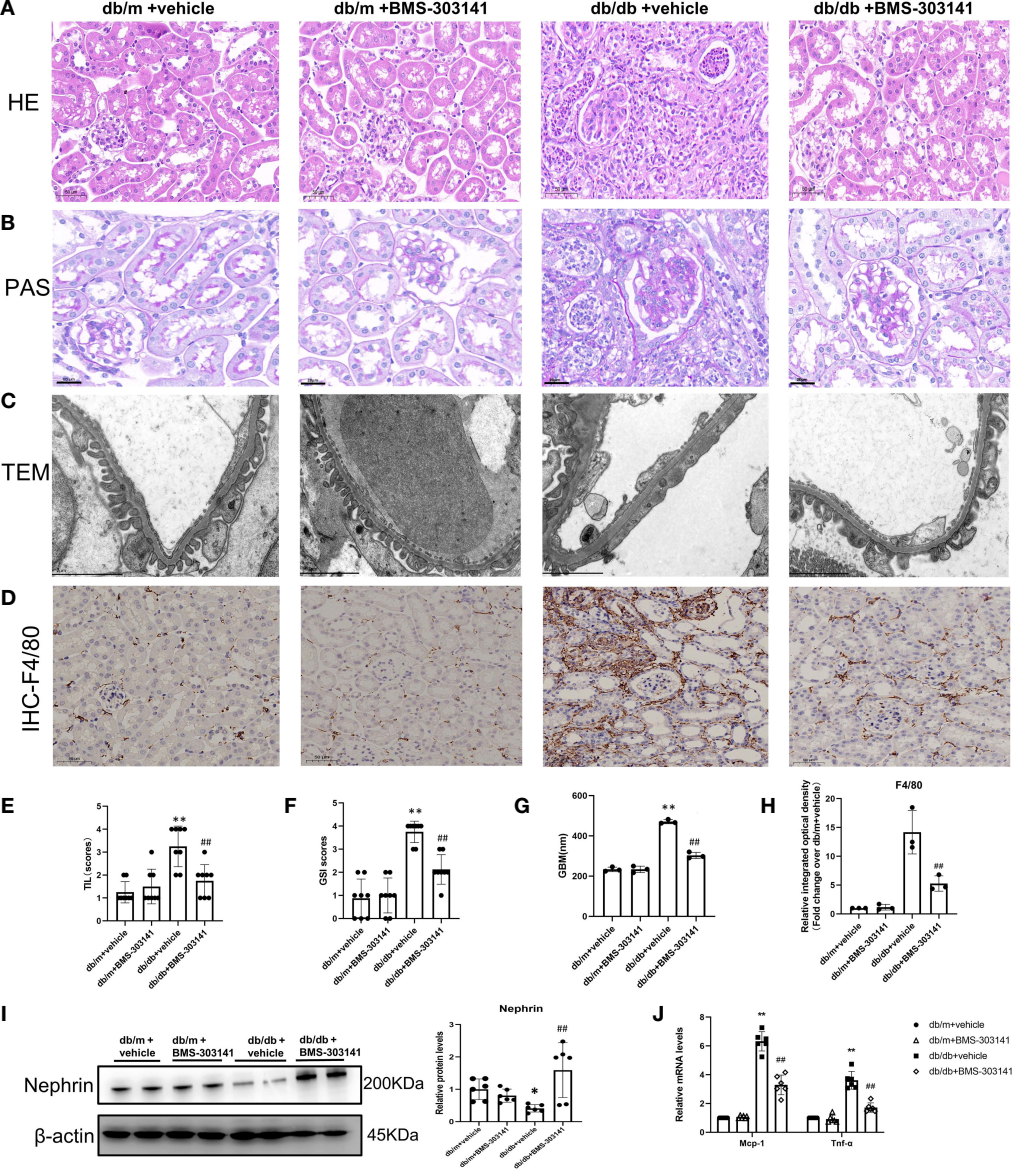
Impact of BMS-303141 on renal histologic injuries. **(A)** Representative H&E staining of kidney cortex sections (magnified 200x) from db/m and db/db mice for 16 weeks; **(B)** Periodic acid-Schiff (PAS)-stained kidney cortex sections (magnification 200x) from db/m and db/db mice at the end of 16 weeks; **(C)** Transmission electron microscopic evaluation of the glomerular filtration barrier of db/m and db/db mice (magnification 7000x), which indicates areas of podocyte foot-process effacement; **(D)** Representative photomicrographs (magnification 200x) illustrating the infiltration of F4/80-positive macrophages in the tissues of the kidneys of mice in various groups; **(E, F)** tubulointerstitial injuries and the level of glomerulosclerosis index were evaluated semiquantitatively as per the Materials and methods section. *P < 0.05, **P < 0.01 versus db/m + vehicle group; #P < 0.05, ##P < 0.01 versus db/db + vehicle group, n = 8 per each group; **(G)** GBM, glomerular basement membrane, and thickness in db/m and db/db mice at the end of 16 weeks. **P < 0.01 versus db/m + vehicle group; ##P < 0.01 versus db/db + vehicle group, n = 3 per each group; **(H)** Semiquantitative immunohistochemical evaluation of the F4/80 inflammation-associated protein expression in various groups. ##P < 0.01 versus db/db + vehicle group, n = 3 per each group; **(I)** Western blots and densitometric quantitation of the indicated protein of the renal damage marker, nephrin, in the kidney cortex from db/m and db/db mice; *P < 0.05 versus db/m + vehicle; ##P < 0.01 versus db/db + vehicle group, n = 6 per each group; **(J)** Quantitative real-time PCR analysis of *Mcp-1*,*Tnf-α* transcripts in the kidney cortex of db/m and db/db mice, *P < 0.05, **P < 0.01 versus db/m + vehicle; #P < 0.05, ##P < 0.01 versus db/db + vehicle group, n = 6 per each group. The mean ± SD is represented by each bar in the four independent experiments.

Furthermore, to ascertain the function of ACL in renal injuries, we focused on the effect of BMS-303141 on renal injuries and inflammation. In comparison to db/m groups, db/db mice had obvious renal injuries, such as glomerular hypertrophy and GBM rupture, glomerulosclerosis, interstitial inflammatory cell infiltration, and tubular dilatation, which were attenuated by BMS-303141 (**Figure 5A-H**). Treatment with BMS-303141 substantially improved the expression of nephrin and glomerular injuries (**Figure 5I**).

Both H&E and IHC staining (F4/80) showed that a large number of macrophages and neutrophils infiltrated the tubular interstitium of lipid-deposited kidneys compared with normal renal tissue (**Figure 5A, D**). Administering BMS-303141 attenuated the inflammatory cell recruitment and pro-inflammatory factor monocyte chemoattractant protein-1 (MCP-1), and TNF-α, the key inflammatory factor in obesity-associated inflammation (**Figure 5J**).

### Tubulointerstitial fibrosis suppressed by BMS-303141 in the kidneys of db/db mice

**Figure 6:**
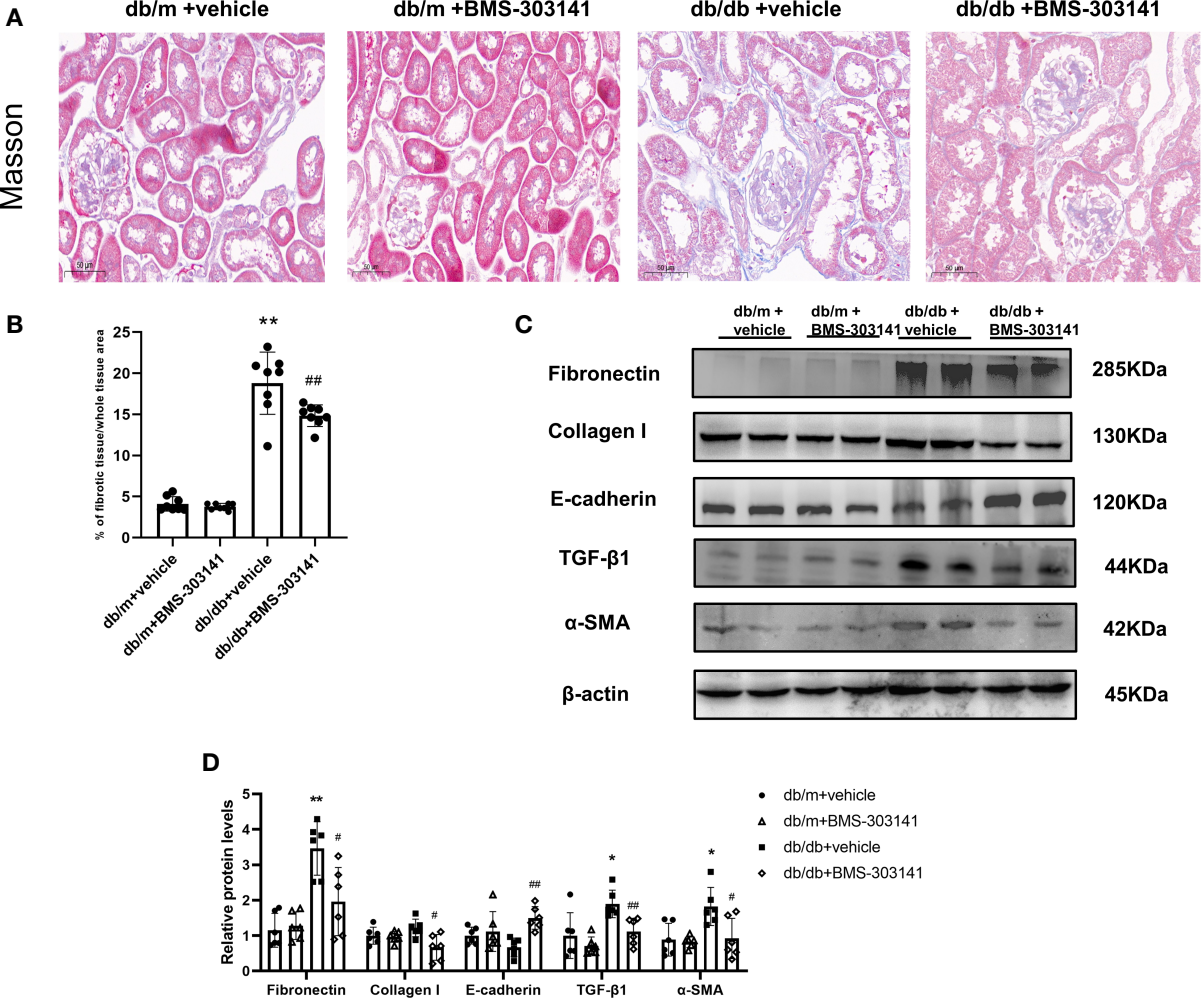
In the kidney tissues of db/db mice, BMS-303141 decreases renal interstitial fibrosis. **(A)** Photomicrographs of renal cortex slices stained with Masson’s trichrome per each group; **(B)** As indicated in the Materials and methods section, the level of tubulointerstitial collagen deposition was measured semiquantitatively. *P < 0.05, **P < 0.01 versus db/m + vehicle group; #P < 0.05, ##P < 0.01 versus db/db + vehicle group, n = 8 per each group; **(C, D)** Western blots **(C)** and densitometric quantitation **(D)** of the indicated protein of renal fibrosis markers, Fibronectin, Collagen I, E-cadherin, TGF-β1, and α-SMA in the kidney cortex from db/m and db/db mice; *P < 0.05, **P < 0.01 versus db/m + vehicle; #P < 0.05, ##P < 0.01, versus db/db + vehicle group, n = 6 per each group. The mean ± SD of four different tests is represented by each bar.

Next, we evaluated the effect of BMS-303141 on epithelial-mesenchymal transition (EMT) and fibrosis after inflammatory reactions. Masson staining showed BMS-303141 treatment reduced the level of tubulointerstitial fibrosis (TIF) which was obvious in db/db mice (**Figure 6A, B**). When comparing treated and untreated db/db mice, E-cadherin protein expression increased, as did α-SMA, Collagen I, TGF-β1, and fibronectin protein expression, which were reversed by administering BMS-303141 (**Figure 6C, D**). These findings imply that BMS-303141 works to prevent fibrosis by inhibiting EMT progression.

## Discussion

In this study, we present evidence that ACL could be a potential therapeutic target in CKD therapy and other kidney diseases that have obesity-related inflammation etiologies. It revealed that the suppression of ACL by BMS-303141 directly inhibited ACL expression and attenuated its effects by preventing renal lipid accumulation and renal injuries in obese mice. Using lipidomics, we found that as the expression of lipogenic genes decreased, the deposition of fatty acids in the kidney changed.

It is reported that the use of microRNAs, small interfering RNAs, and ACL inhibitors which consider ACL as the target has provided a promising therapeutic insight for the treatment of chronic diseases (25). Therefore, we hope to find a better ACL inhibitor by molecular docking for obesity-related kidney disease. As seen in **Table 2**, among the binding energies for the four docked complex crystal structures, BMS-303141 showed a binding energy > −8 kcal/mol and RMSD <4 Å, indicating that it had the highest affinity for the specific protein. Normally, the effect of small molecule inhibitors is through binding directly to the target protein, competing with the substrate, altering the protein structure, or hindering the conformational transition of the protein configuration, thereby reducing protein activity. Furthermore, regarding the downregulation of ACL in db/db mice treated with BMS-303141, it could have resulted from protein accumulation, which may cause negative feedback leading to reduced ACL biosynthesis. And in a previous study, we showed that inflammation could stimulate ACL expression through the NF-κB pathway (13, 14). Thus, BMS-303141 may downregulate ACL protein expression in the db/db mouse group by attenuating the inflammatory process.

ACL is found to be a critical mediator of aerobic glycolysis and *de novo* lipid production in multiple types of malignant cells. It has been noted that lipid synthesis increasing as one of the key characteristics of many cancers is critical for cancer progression (26). So, decreasing the expression of fatty acid enzymes, such as ACL and FAS, resulted in the inhibition of growth and the proliferation of tumor cells (27). In db/db mice, ACL is highly induced in the kidney and is correlated with renal ELA, albuminuria, and glomerulosclerosis. The positive correlation between the renal expression of ACL and the urinary albumin to creatinine ratio and renal nephrin demonstrated that ACL plays an essential function in the development of albuminuria. ACL expression was inhibited after BMS-303141 administration, as was the downregulated expression of enzyme proteins associated with fatty acid and cholesterol biosynthesis, ACC, FAS, and HMGCR. This means the histone acetylation resulting from ACL could be downregulated by BMS-303141 by decreasing the supply of acetyl-CoA as a substrate. This study revealed that the suppression of ACL by BMS-303141 directly inhibited ACL expression and mitigated its effects by preventing renal lipid accumulation and renal cell injuries in obese mice. However, the hyperglycemic state was not attenuated. This could be because the dosage used in the experiment could not reverse the serious consequences of pancreatic dysfunction or the degeneration of islet cells. In a high-fat diet mouse model, administering 10 mg/kg/day BMS-303141 for 29 days reduced plasma glucose (9).

Our experiments to prove the effectiveness of the inhibitor molecules and to explore the effect of ACL inhibition on the metabolic pathway showing statistically significant lipid species in both treated and untreated db/db groups. We found differences in predominant lipid species, especially in glyceride and fatty acid, revealing the level of ectopic lipid deposition in the kidney that caused obesity-associated inflammation, resulting in the progression of obesity-related CKD. Unfortunately, the content of sterol lipids was not detected by mass spectrometry, which means we cannot tell the trends of cholesterol in the kidneys. The main metabolites are sphingolipids, including the two central bioactive lipids ceramide and sphingosine-1-phosphate, and the function of sphingolipid rebiosynthesis is widespread within cells and tissues. Ceramide has various biological functions, including inducing apoptosis, regulating cell differentiation and growth, and modulating immune function and inflammation. Conversely, cellular lipid accumulation may cause the secretion of cytokines like Interleukin-6 and TNF-α, which promote lipolysis and lead to ceramide accumulation. Feeding rats with a high-fat diet for 3 weeks reportedly caused an accumulation of ceramide and sphingomyelin in the nucleus, and eventually a fatty liver (28). In this research, BMS-303141 decreased cellular lipid accumulation, which reduced the recruitment of cytokines like TNF-α and TGF-β, and lowered damage from ceramide accumulation. Therefore, BMS-303141 may affect sphingolipids by inhibiting lipid accumulation and inflammation. The decrease in glyceride may suggest that the lipocatabolism and insulin resistence could be attenuated in kidney.

Research showed that ACL suppression could diminish the production of nitric oxide, reactive oxygen species, and prostaglandin E2, thereby alleviating inflammation in Down syndrome (29). In obesity-related renal injuries, ELA is deemed to be a critical cause oflipotoxicity in the kidneys (3, 6), and it finally stimulates pro-inflammatory and profibrogenic pathways (7). Several studies have shown that obesity-associated inflammation stimulates pro-inflammatory cytokines such as TNF-α or Interleukin-1, which can trigger EMT in the epithelial cells (30). Additionally, TGF-β1 has also been found to be the major inducer of EMT and consequent interstitial ECM synthesis in the kidneys and various organ systems (31, 32). Infiltrating macrophages in mouse kidneys typically correlates with the degree of renal fibrosis (22, 33). Our research showed BMS-303141 inhibited the infiltration of macrophages and the release of pro-inflammatory chemokine (TNF-α, MCP-1). Meanwhile, the molecule may also block the tubular EMT process and tubulointerstitial fibrosis, which are possibly mediated through the overproduction of TGF-β1.

To the best of our knowledge, it is the first time that the antifibrotic and anti-ELA effect of ACL inhibitors has been determined using the db/db mouse model and the feasibility of ACL as a therapeutic target for obesity-related CKD has been investigated. We found that BMS-303141 attenuated tubular injuries, ECM protein accumulation, and ectopic lipid deposition in the kidneys of obese mice. We also found that BMS-303141 inhibited inflammation and fibrosis in the kidney of db/db mice. BMS-303141 not only reduced the expression of lipogenic enzymes and fibrogenes directly by reducing the content of acetyl-CoA as the substrate for histone acetylation but also affected the TCA cycle by decreasing the biosynthesis of fatty acids. These results might be utilized for further investigations to ascertain the therapeutic benefit of ACL in attenuating progressive obesity-related CKD. Finally, the lipidomic analysis not only showed the lipid-lowering effect of BMS-303141 in the kidney but also suggested that ACL could affect the sphingolipid metabolism pathway, which has not been investigated. Future investigations are necessary to explore the interaction between ACL and sphingolipid metabolism and the functional role of upregulated or downregulated ACL expression in changes in the cell membrane or other biomembrane structures. This will further advance our comprehension of the function of the ACL protein in the impairment of kidney disease and guide potential therapies.

## Data availability statement

The original contributions presented in the study are included in the article/**Supplementary Material**. Further inquiries can be directed to the corresponding authors.

## Ethics statement

The animal study was reviewed and approved by Institutional Animal Care and Use Committee (IACUC) of Central South University (Approval no. CSU-2022-0001-0311).

## Author contributions

JS, ZZ, and AL contributed to the study design. All authors contributed to writing the manuscript and approved the final versionJS, YL, HZ conceived the study. All authors contributed to the article and approved the submitted version.

## Supplementary material

Supplementary Figure 1 (A) Oil Red O staining of renal cortex slices from db/m and db/db mice for 12 and 16 weeks (magnifying 400x); (B) photomicrographs of renal cortex slices stained with Masson’s trichrome (magnifying 300x) from 12- and 16-week-old db/m and db/db mice; (C) representative photomicrographs (magnification 200x) illustrating the infiltration of ACL-positive areas in the kidney tissues in each of the 16 weeks db/db mouse groups.

Supplementary Figure 2 Mechanism graph, summed up from Professor Li’s previous studies (9). In glycometabolism disease, hyperglycemia and hyperlipidemia promote nutritional over-influx into cells, resulting in increased TCA cycle product and elevated citrate and ATP levels. In many tissues, ACL is the major enzyme that converts citrate and CoA into cytosolic acetyl-CoA. And the overproduced ATP and inflammation co-stimulate ACL expression *via* the cAMP-PKA pathway and NF-κB pathway, respectively. Then, acetyl-CoA is not only presented as the substrate of ELA but also the source of histone acetylation, which results in the upregulation of the epigenetic process of ELA-related and profibrotic genes in mesangial cells, such as ACC, FAS, HMGCR, TGF-β1[17]. TGF-1 enhances renal fibrogenesis, and overexpression of ACC, FAS and HMGCR promotes *de novo* lipogenesis from excessive acetyl-CoA in the cytosol, which contributes to renal ELA exacerbation (9). In *in vivo* experiment, we ascertained the anti-fibrotic and anti-ELA effects of BMS-303141 using the db/db mouse model. We discovered that the level of ELA and fibrosis in the kidney was reduced by ACL inhibitor administration. This not only reduced the expression of lipogenic enzymes and fibrogenes directly by attenuating histone acetylation but also relieved the TCA cycle by decreasing fatty acid production.

Supplementary Table 1 The differential lipid metabolites Group D vs. Group C. PE, phosphatid ylethanolamine; CL, Cardiolipin; DGDG, Digalactosyldiacylglycerol; dMePE, dimethylphosphatidylethanolamine; LPG,lysophospha tidylglycerol; PC, phosphatidylcholine; PG, phosphatidylglycerol; PI, phosphatidylinositol; PS, phosphatidylserine; SM, sphingomyelin; Cer, Ceramides; CerG1, Simple Glc series; CerG2, Simple Glc series; DG, diglyceride; LPC, lysophosphatidylcholine; LPet, lysophosp hatidylethanol; PhSM, sphingomyelin(phytosphingosine); So, Sphingosine; TG, triglyceride; LdMePE, lysodimethylphosphatidylethanolamine; LPE, lysophosphatidylethanolamine; LPI, lysophosphatidylinositol; LPS, lysophosphatidylserine; MGDG, Digalactosylmonoacylglycerol; SQDG, Sulfoquinovosyldiacylglycerol; MG, monoglyceride. PA, phosphatidic acid.
