# Supplementary figures and images for: ATP-citrate lyase inhibitor improves ectopic lipid accumulation in the kidney in a db/db mouse model
Source: Front Endocrinol (Lausanne). 2022 Dec 8;13:914865. doi: 10.3389/fendo.2022.914865 (PMC9771989; doi:10.3389/fendo.2022.914865)

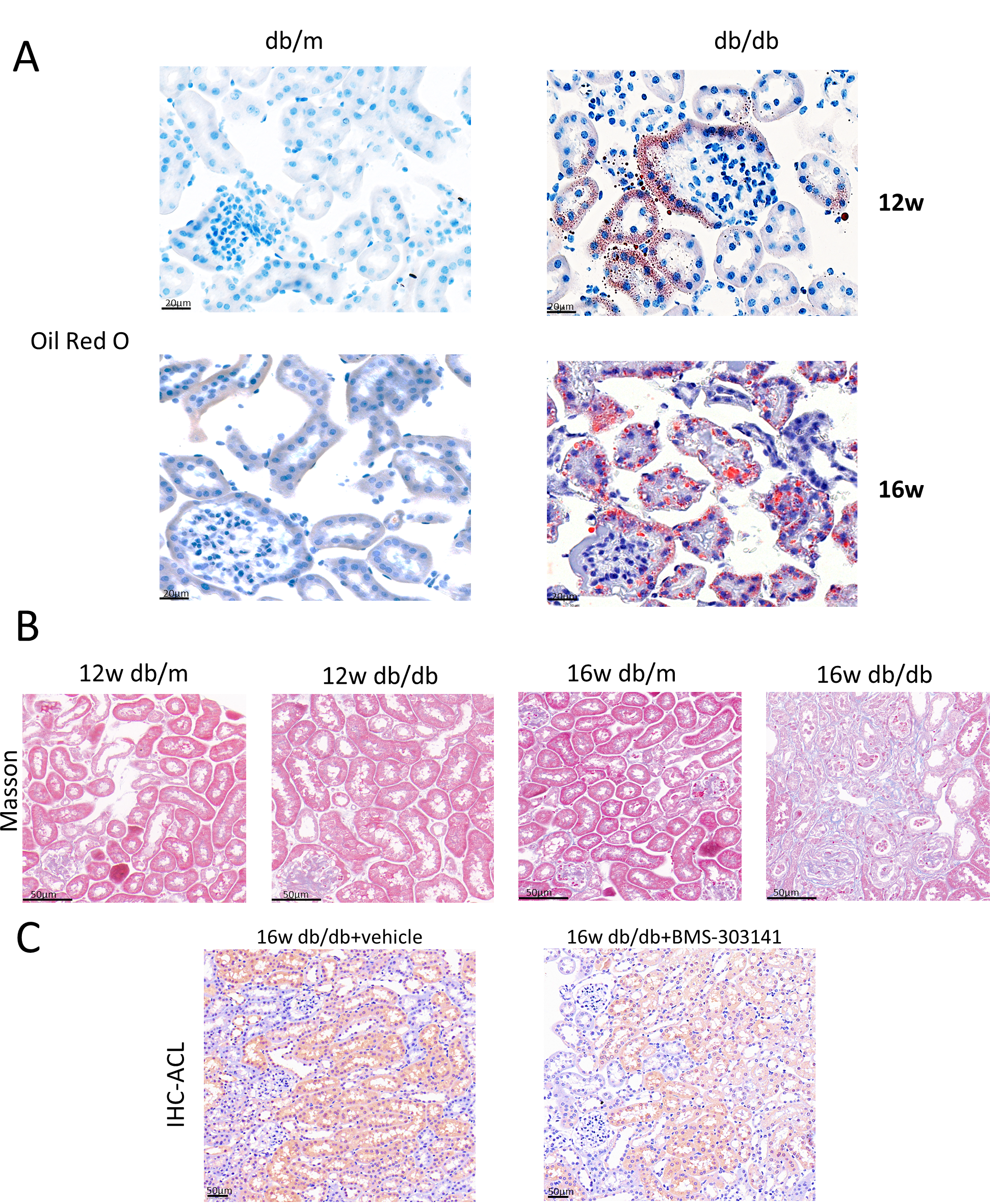

Supplement: Supplementary Figure 1 — (A) Oil Red O staining of renal cortex slices from db/m and db/db mice for 12 and 16 weeks (magnifying 400x); (B) photomicrographs of renal cortex slices stained with Masson’s trichrome (magnifying 300x) from 12- and 16-week-old db/m and db/db mice; (C) representative photomicrographs (magnification 200x) illustrating the infiltration of ACL-positive areas in the kidney tissues in each of the 16 weeks db/db mouse groups. [file Image_1.tif]

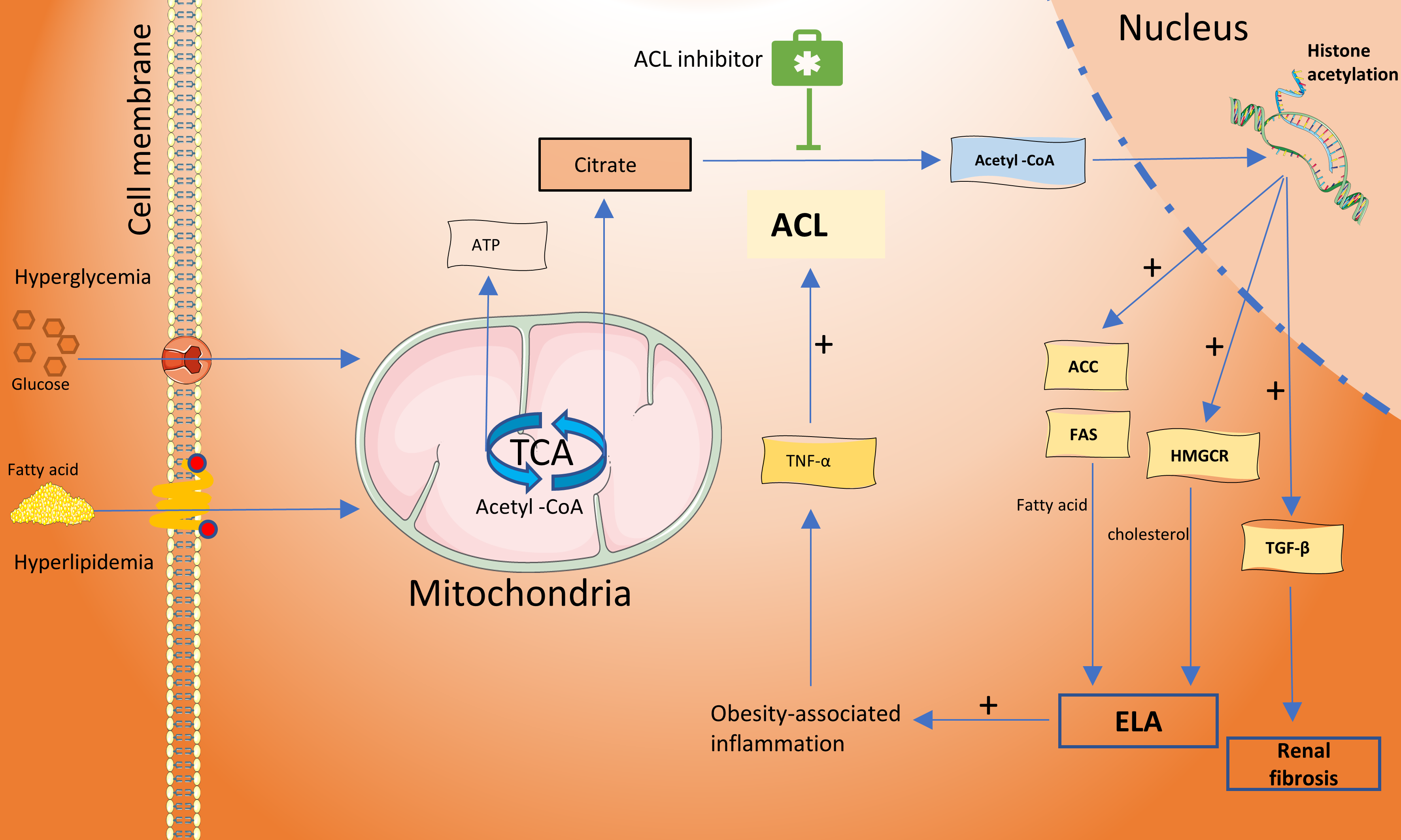

Supplement: Supplementary Figure 2 — Mechanism graph, summed up from Professor Li’s previous studies (9). In glycometabolism disease, hyperglycemia and hyperlipidemia promote nutritional over-influx into cells, resulting in increased TCA cycle product and elevated citrate and ATP levels. In many tissues, ACL is the major enzyme that converts citrate and CoA into cytosolic acetyl-CoA. And the overproduced ATP and inflammation co-stimulate ACL expression via the cAMP-PKA pathway and NF-κB pathway, respectively. Then, acetyl-CoA is not only presented as the substrate of ELA but also the source of histone acetylation, which results in the upregulation of the epigenetic process of ELA-related and profibrotic genes in mesangial cells, such as ACC, FAS, HMGCR, TGF-β1[17]. TGF-1 enhances renal fibrogenesis, and overexpression of ACC, FAS and HMGCR promotes de novo lipogenesis from excessive acetyl-CoA in the cytosol, which contributes to renal ELA exacerbation (9). In in vivo experiment, we ascertained the anti-fibrotic and anti-ELA effects of BMS-303141 using the db/db mouse model. We discovered that the level of ELA and fibrosis in the kidney was reduced by ACL inhibitor administration. This not only reduced the expression of lipogenic enzymes and fibrogenes directly by attenuating histone acetylation but also relieved the TCA cycle by decreasing fatty acid production. [file Image_2.tif]
