# Supplementary material for: ATP-citrate lyase inhibitor improves ectopic lipid accumulation in the kidney in a db/db mouse model
Source: Front Endocrinol (Lausanne). 2022 Dec 8;13:914865. doi: 10.3389/fendo.2022.914865 (PMC9771989; doi:10.3389/fendo.2022.914865)
Supplement: Supplementary Table 1 — The differential lipid metabolites Group D vs. Group C. PE, phosphatid ylethanolamine; CL, Cardiolipin; DGDG, Digalactosyldiacylglycerol; dMePE, dimethylphosphatidylethanolamine; LPG,lysophospha tidylglycerol; PC, phosphatidylcholine; PG, phosphatidylglycerol; PI, phosphatidylinositol; PS, phosphatidylserine; SM, sphingomyelin; Cer, Ceramides; CerG1, Simple Glc series; CerG2, Simple Glc series; DG, diglyceride; LPC, lysophosphatidylcholine; LPet, lysophosp hatidylethanol; PhSM, sphingomyelin(phytosphingosine); So, Sphingosine; TG, triglyceride; LdMePE, lysodimethylphosphatidylethanolamine; LPE, lysophosphatidylethanolamine; LPI, lysophosphatidylinositol; LPS, lysophosphatidylserine; MGDG, Digalactosylmonoacylglycerol; SQDG, Sulfoquinovosyldiacylglycerol; MG, monoglyceride. PA, phosphatidic acid. [file Table_1.docx]

Supplement Table. 1 The differential lipid metabolites Group D vs. Group C

| LipidIon | Adduction | LipidGroup | Class |
| --- | --- | --- | --- |
| CL(18:1/16:0/16:0/16:0)-H | M-H | CL(66:1) | CL |
| CL(18:1/16:0/18:1/16:0)-H | M-H | CL(68:2) | CL |
| DGDG(16:0/16:0)+HCOO | M+HCOO | DGDG(32:0) | DGDG |
| dMePE(16:0e/22:6)-H | M-H | dMePE(38:6e) | dMePE |
| dMePE(18:2p/22:6)-H | M-H | dMePE(40:8p) | dMePE |
| dMePE(34:0p)-H | M-H | dMePE(34:0p) | dMePE |
| dMePE(38:6p)-H | M-H | dMePE(38:6p) | dMePE |
| LPG(22:6)-H | M-H | LPG(22:6) | LPG |
| PA(16:0/22:6)-H | M-H | PA(38:6) | PA |
| PC(14:0e/16:0)+HCOO | M+HCOO | PC(30:0e) | PC |
| PC(14:0e/22:6)+HCOO | M+HCOO | PC(36:6e) | PC |
| PC(16:0e/16:0)+HCOO | M+HCOO | PC(32:0e) | PC |
| PC(16:1p/22:6)+HCOO | M+HCOO | PC(38:7p) | PC |
| PC(18:0e/16:0)+HCOO | M+HCOO | PC(34:0e) | PC |
| PC(20:5/22:6)+HCOO | M+HCOO | PC(42:11) | PC |
| PC(22:5/22:6)+HCOO | M+HCOO | PC(44:11) | PC |
| PC(22:6/22:6)+HCOO | M+HCOO | PC(44:12) | PC |
| PE(16:0/22:6)-H | M-H | PE(38:6) | PE |
| PE(18:1p/22:5)-H | M-H | PE(40:6p) | PE |
| PE(34:0e)-H | M-H | PE(34:0e) | PE |
| PG(16:0/22:6)-H | M-H | PG(38:6) | PG |
| PI(16:0/16:0)-H | M-H | PI(32:0) | PI |
| PI(16:0/22:6)-H | M-H | PI(38:6) | PI |
| PI(16:0e/22:6)-H | M-H | PI(38:6e) | PI |
| PS(16:0/22:6)-H | M-H | PS(38:6) | PS |
| PS(18:0/22:6)-H | M-H | PS(40:6) | PS |
| PS(18:1/22:6)-H | M-H | PS(40:7) | PS |
| PS(40:8)-H | M-H | PS(40:8) | PS |
| PS(40:9)-H | M-H | PS(40:9) | PS |
| PS(45:8)-H | M-H | PS(45:8) | PS |
| SM(d34:1)+CH3COO | M+CH3COO | SM(d34:1) | SM |
| Cer(d26:0)+H | M+H | Cer(d26:0) | Cer |
| Cer(d28:0)+H | M+H | Cer(d28:0) | Cer |
| Cer(d30:0)+H | M+H | Cer(d30:0) | Cer |
| Cer(d30:0+O)+H | M+H | Cer(d30:0+O) | Cer |
| Cer(d32:0)+H | M+H | Cer(d32:0) | Cer |
| Cer(d18:2/16:1)+H | M+H | Cer(d34:3) | Cer |
| Cer(d36:0)+H | M+H | Cer(d36:0) | Cer |
| Cer(d38:1)+H | M+H | Cer(d38:1) | Cer |
| Cer(d38:3)+H | M+H | Cer(d38:3) | Cer |
| Cer(d18:1/24:1)+H | M+H | Cer(d42:2) | Cer |
| CerG1(d18:1/22:1)+H | M+H | CerG1(d40:2) | CerG1 |
| CerG1(d17:0/26:2)+H | M+H | CerG1(d43:2) | CerG1 |
| CerG2(d32:0)+Na | M+Na | CerG2(d32:0) | CerG2 |
| DG(18:0/18:0)+NH4 | M+NH4 | DG(36:0) | DG |
| LPC(19:0)+H | M+H | LPC(19:0) | LPC |
| LPC(20:0)+H | M+H | LPC(20:0) | LPC |
| LPC(22:3)+H | M+H | LPC(22:3) | LPC |
| LPC(24:0)+H | M+H | LPC(24:0) | LPC |
| LPC(32:0)+Na | M+Na | LPC(32:0) | LPC |
| LPC(34:1)+Na | M+Na | LPC(34:1) | LPC |
| LPC(36:6)+Na | M+Na | LPC(36:6) | LPC |
| LPC(37:6)+H | M+H | LPC(37:6) | LPC |
| LPG(20:3)+Na | M+Na | LPG(20:3) | LPG |
| LPG(22:6)+H | M+H | LPG(22:6) | LPG |
| LPG(22:6)+Na | M+Na | LPG(22:6) | LPG |
| PC(21:0e)+H | M+H | PC(21:0e) | PC |
| PC(23:0e)+H | M+H | PC(23:0e) | PC |
| PC(30:0)+H | M+H | PC(30:0) | PC |
| PC(30:0e)+H | M+H | PC(30:0e) | PC |
| PC(31:0)+H | M+H | PC(31:0) | PC |
| PC(31:0e)+H | M+H | PC(31:0e) | PC |
| PC(16:0/16:0)+Na | M+Na | PC(32:0) | PC |
| PC(32:0e)+H | M+H | PC(32:0e) | PC |
| PC(32:0e)+Na | M+Na | PC(32:0e) | PC |
| PC(32:1e)+H | M+H | PC(32:1e) | PC |
| PC(34:0e)+H | M+H | PC(34:0e) | PC |
| PC(34:0e)+Na | M+Na | PC(34:0e) | PC |
| PC(16:0p/18:0)+Na | M+Na | PC(34:0p) | PC |
| PC(34:1e)+H | M+H | PC(34:1e) | PC |
| PC(34:1e)+Na | M+Na | PC(34:1e) | PC |
| PC(34:3p)+H | M+H | PC(34:3p) | PC |
| PC(34:4p)+H | M+H | PC(34:4p) | PC |
| PC(35:4)+H | M+H | PC(35:4) | PC |
| PC(36:0e)+H | M+H | PC(36:0e) | PC |
| PC(36:3)+Na | M+Na | PC(36:3) | PC |
| PC(16:0/20:4)+Na | M+Na | PC(36:4) | PC |
| PC(36:5)+H | M+H | PC(36:5) | PC |
| PC(16:1p/20:4)+Na | M+Na | PC(36:5p) | PC |
| PC(36:6)+Na | M+Na | PC(36:6) | PC |
| PC(36:6p)+H | M+H | PC(36:6p) | PC |
| PC(36:6p)+Na | M+Na | PC(36:6p) | PC |
| PC(36:7p)+H | M+H | PC(36:7p) | PC |
| PC(37:3)+H | M+H | PC(37:3) | PC |
| PC(37:6)+H | M+H | PC(37:6) | PC |
| PC(37:6p)+H | M+H | PC(37:6p) | PC |
| PC(37:7)+H | M+H | PC(37:7) | PC |
| PC(37:9)+H | M+H | PC(37:9) | PC |
| PC(38:3)+Na | M+Na | PC(38:3) | PC |
| PC(38:6)+H | M+H | PC(38:6) | PC |
| PC(38:6e)+Na | M+Na | PC(38:6e) | PC |
| PC(38:6p)+Na | M+Na | PC(38:6p) | PC |
| PC(38:7)+Na | M+Na | PC(38:7) | PC |
| PC(38:8)+H | M+H | PC(38:8) | PC |
| PC(39:1p)+H | M+H | PC(39:1p) | PC |
| PC(39:2)+H | M+H | PC(39:2) | PC |
| PC(39:6)+H | M+H | PC(39:6) | PC |
| PC(39:6p)+Na | M+Na | PC(39:6p) | PC |
| PC(39:8)+H | M+H | PC(39:8) | PC |
| PC(17:4/22:5)+H | M+H | PC(39:9) | PC |
| PC(40:11)+H | M+H | PC(40:11) | PC |
| PC(40:6)+H | M+H | PC(40:6) | PC |
| PC(40:6)+Na | M+Na | PC(40:6) | PC |
| PC(40:6e)+H | M+H | PC(40:6e) | PC |
| PC(40:6p)+Na | M+Na | PC(40:6p) | PC |
| PC(40:7p)+H | M+H | PC(40:7p) | PC |
| PC(40:7p)+Na | M+Na | PC(40:7p) | PC |
| PC(16:2/24:6)+Na | M+Na | PC(40:8) | PC |
| PC(40:8p)+Na | M+Na | PC(40:8p) | PC |
| PC(41:6)+H | M+H | PC(41:6) | PC |
| PC(42:10)+Na | M+Na | PC(42:10) | PC |
| PC(42:11)+Na | M+Na | PC(42:11) | PC |
| PC(42:4p)+H | M+H | PC(42:4p) | PC |
| PC(42:5)+Na | M+Na | PC(42:5) | PC |
| PC(42:6e)+H | M+H | PC(42:6e) | PC |
| PC(42:7e)+H | M+H | PC(42:7e) | PC |
| PC(42:7p)+H | M+H | PC(42:7p) | PC |
| PC(42:8p)+H | M+H | PC(42:8p) | PC |
| PC(42:9)+H | M+H | PC(42:9) | PC |
| PC(44:10)+H | M+H | PC(44:10) | PC |
| PC(44:11)+H | M+H | PC(44:11) | PC |
| PC(44:11)+Na | M+Na | PC(44:11) | PC |
| PC(44:12)+H | M+H | PC(44:12) | PC |
| PC(44:12)+Na | M+Na | PC(44:12) | PC |
| PC(44:4)+H | M+H | PC(44:4) | PC |
| PC(46:12)+H | M+H | PC(46:12) | PC |
| PC(46:6)+H | M+H | PC(46:6) | PC |
| PE(34:2e)+H | M+H | PE(34:2e) | PE |
| PE(36:1)+Na | M+Na | PE(36:1) | PE |
| PE(16:0/20:5)+H | M+H | PE(36:5) | PE |
| PE(14:0p/22:6)+H | M+H | PE(36:6p) | PE |
| PE(37:0p)+H | M+H | PE(37:0p) | PE |
| PE(37:0p)+Na | M+Na | PE(37:0p) | PE |
| PE(38:3)+Na | M+Na | PE(38:3) | PE |
| PE(38:4)+Na | M+Na | PE(38:4) | PE |
| PE(38:6)+Na | M+Na | PE(38:6) | PE |
| PE(16:0e/22:6)+Na | M+Na | PE(38:6e) | PE |
| PE(16:0p/22:6)+Na | M+Na | PE(38:6p) | PE |
| PE(22:5/17:2)+H | M+H | PE(39:7) | PE |
| PE(40:3)+Na | M+Na | PE(40:3) | PE |
| PE(20:1/20:4)+H | M+H | PE(40:5) | PE |
| PE(40:6)+H | M+H | PE(40:6) | PE |
| PE(40:6)+Na | M+Na | PE(40:6) | PE |
| PE(40:6p)+Na | M+Na | PE(40:6p) | PE |
| PE(18:1/22:6)+H | M+H | PE(40:7) | PE |
| PE(18:1p/22:6)+H | M+H | PE(40:7p) | PE |
| PE(40:7p)+Na | M+Na | PE(40:7p) | PE |
| PE(40:8)+Na | M+Na | PE(40:8) | PE |
| PE(18:2p/22:6)+H | M+H | PE(40:8p) | PE |
| PE(41:6e)+H | M+H | PE(41:6e) | PE |
| PE(42:2)+H | M+H | PE(42:2) | PE |
| PE(42:2e)+H | M+H | PE(42:2e) | PE |
| PE(20:0p/22:6)+H | M+H | PE(42:6p) | PE |
| PE(42:7e)+Na | M+Na | PE(42:7e) | PE |
| PE(20:1p/22:6)+H | M+H | PE(42:7p) | PE |
| PE(43:7p)+Na | M+Na | PE(43:7p) | PE |
| PE(47:3)+H | M+H | PE(47:3) | PE |
| PE(62:6)+H | M+H | PE(62:6) | PE |
| PEt(33:0)+Na | M+Na | PEt(33:0) | PEt |
| PG(22:6/22:6)+H | M+H | PG(44:12) | PG |
| PG(22:6/22:6)+Na | M+Na | PG(44:12) | PG |
| PG(22:6/22:6)+NH4 | M+NH4 | PG(44:12) | PG |
| phSM(d40:1)+H | M+H | phSM(d40:1) | phSM |
| phSM(d40:6)+H | M+H | phSM(d40:6) | phSM |
| phSM(d41:1)+H | M+H | phSM(d41:1) | phSM |
| phSM(d42:6)+H | M+H | phSM(d42:6) | phSM |
| phSM(d42:8)+H | M+H | phSM(d42:8) | phSM |
| phSM(d54:3)+H | M+H | phSM(d54:3) | phSM |
| phSM(d56:3)+H | M+H | phSM(d56:3) | phSM |
| PI(16:0/20:3)+Na | M+Na | PI(36:3) | PI |
| PI(36:4)+NH4 | M+NH4 | PI(36:4) | PI |
| PI(36:4e)+H | M+H | PI(36:4e) | PI |
| PI(16:0/22:6)+H | M+H | PI(38:6) | PI |
| PI(38:6)+Na | M+Na | PI(38:6) | PI |
| PI(16:0/22:6)+NH4 | M+NH4 | PI(38:6) | PI |
| PS(38:4)+Na | M+Na | PS(38:4) | PS |
| PS(18:1/20:5)+H | M+H | PS(38:6) | PS |
| PS(38:6)+Na | M+Na | PS(38:6) | PS |
| PS(40:5)+H | M+H | PS(40:5) | PS |
| PS(40:6)+Na | M+Na | PS(40:6) | PS |
| PS(18:1/22:6)+H | M+H | PS(40:7) | PS |
| SM(d34:2)+Na | M+Na | SM(d34:2) | SM |
| SM(d35:1)+Na | M+Na | SM(d35:1) | SM |
| SM(d38:1)+Na | M+Na | SM(d38:1) | SM |
| SM(d41:2)+Na | M+Na | SM(d41:2) | SM |
| SM(d43:1)+Na | M+Na | SM(d43:1) | SM |
| SM(d43:2)+Na | M+Na | SM(d43:2) | SM |
| SM(d18:1/25:4)+H | M+H | SM(d43:5) | SM |
| SM(d44:1)+H | M+H | SM(d44:1) | SM |
| SM(d44:2)+H | M+H | SM(d44:2) | SM |
| SM(d45:5)+H | M+H | SM(d45:5) | SM |
| So(d14:0)+H | M+H | So(d14:0) | So |
| TG(18:0/22:6/22:6)+NH4 | M+NH4 | TG(62:12) | TG |
| Cer(d18:1/22:0)-H | M-H | Cer(d40:1) | Cer |
| dMePE(18:1/18:1)-H | M-H | dMePE(36:2) | dMePE |
| dMePE(40:5p)-H | M-H | dMePE(40:5p) | dMePE |
| LdMePE(18:2)-H | M-H | LdMePE(18:2) | LdMePE |
| LPC(16:1)+HCOO | M+HCOO | LPC(16:1) | LPC |
| LPC(18:2)+HCOO | M+HCOO | LPC(18:2) | LPC |
| LPE(18:2)-H | M-H | LPE(18:2) | LPE |
| LPG(18:1)-H | M-H | LPG(18:1) | LPG |
| LPG(18:2)-H | M-H | LPG(18:2) | LPG |
| LPI(18:1)-H | M-H | LPI(18:1) | LPI |
| LPS(18:1)-H | M-H | LPS(18:1) | LPS |
| LPS(18:2)-H | M-H | LPS(18:2) | LPS |
| MGDG(37:7)+HCOO | M+HCOO | MGDG(37:7) | MGDG |
| PC(16:1/18:2)+HCOO | M+HCOO | PC(34:3) | PC |
| PC(18:2/18:2)+HCOO | M+HCOO | PC(36:4) | PC |
| PE(17:0/18:2)-H | M-H | PE(35:2) | PE |
| PE(18:0/18:2)-H | M-H | PE(36:2) | PE |
| PG(18:1/18:2)-H | M-H | PG(36:3) | PG |
| PG(18:1/20:3)-H | M-H | PG(38:4) | PG |
| PG(18:1/22:5)-H | M-H | PG(40:6) | PG |
| PG(18:1/22:6)-H | M-H | PG(40:7) | PG |
| PG(18:2/22:6)-H | M-H | PG(40:8) | PG |
| PG(20:1/18:2)-H | M-H | PG(38:3) | PG |
| PG(20:3/18:2)-H | M-H | PG(38:5) | PG |
| PG(22:4/18:2)-H | M-H | PG(40:6) | PG |
| PG(35:1)-H | M-H | PG(35:1) | PG |
| PG(41:8)-H | M-H | PG(41:8) | PG |
| PI(18:0/18:1)-H | M-H | PI(36:1) | PI |
| PI(18:1/18:2)-H | M-H | PI(36:3) | PI |
| PI(18:1/22:5)-H | M-H | PI(40:6) | PI |
| PI(18:2/22:6)-H | M-H | PI(40:8) | PI |
| SQDG(46:8)+HCOO | M+HCOO | SQDG(46:8) | SQDG |
| DG(34:2p)+Na | M+Na | DG(34:2p) | DG |
| DG(18:1/18:1)+H | M+H | DG(36:2) | DG |
| DG(18:1/18:1)+NH4 | M+NH4 | DG(36:2) | DG |
| DG(18:1/18:2)+H | M+H | DG(36:3) | DG |
| DG(18:1/18:2)+NH4 | M+NH4 | DG(36:3) | DG |
| DG(36:4e)+H | M+H | DG(36:4e) | DG |
| DG(40:5e)+H | M+H | DG(40:5e) | DG |
| LPC(15:2)+H | M+H | LPC(15:2) | LPC |
| LPE(18:2)+H | M+H | LPE(18:2) | LPE |
| MG(36:4)+H | M+H | MG(36:4) | MG |
| MG(38:5)+H | M+H | MG(38:5) | MG |
| So(d18:0)+H | M+H | So(d18:0) | So |
| TG(18:1/12:0/12:0)+NH4 | M+NH4 | TG(42:1) | TG |
| TG(12:0/12:0/18:2)+NH4 | M+NH4 | TG(42:2) | TG |
| TG(6:0/18:1/18:2)+NH4 | M+NH4 | TG(42:3) | TG |
| TG(18:1/12:0/14:0)+NH4 | M+NH4 | TG(44:1) | TG |
| TG(16:1/12:0/16:1)+NH4 | M+NH4 | TG(44:2) | TG |
| TG(16:1/10:0/18:2)+NH4 | M+NH4 | TG(44:3) | TG |
| TG(8:0/18:2/18:2)+NH4 | M+NH4 | TG(44:4) | TG |
| TG(15:0/14:0/16:1)+NH4 | M+NH4 | TG(45:1) | TG |
| TG(15:0/12:0/18:2)+NH4 | M+NH4 | TG(45:2) | TG |
| TG(9:0/18:1/18:2)+NH4 | M+NH4 | TG(45:3) | TG |
| TG(16:1/12:0/18:1)+NH4 | M+NH4 | TG(46:2) | TG |
| TG(16:1/12:0/18:2)+NH4 | M+NH4 | TG(46:3) | TG |
| TG(10:0/18:2/18:2)+NH4 | M+NH4 | TG(46:4) | TG |
| TG(10:0/18:2/18:3)+NH4 | M+NH4 | TG(46:5) | TG |
| TG(15:0/16:0/16:1)+NH4 | M+NH4 | TG(47:1) | TG |
| TG(15:0/14:0/18:2)+NH4 | M+NH4 | TG(47:2) | TG |
| TG(15:1/16:1/16:1)+NH4 | M+NH4 | TG(47:3) | TG |
| TG(16:0e/16:0/16:0)+H | M+H | TG(48:0e) | TG |
| TG(16:0e/16:0/16:0)+NH4 | M+NH4 | TG(48:0e) | TG |
| TG(16:0/14:0/18:3)+NH4 | M+NH4 | TG(48:3) | TG |
| TG(12:0/18:2/18:3)+NH4 | M+NH4 | TG(48:5) | TG |
| TG(15:0/16:0/18:1)+NH4 | M+NH4 | TG(49:1) | TG |
| TG(15:0/16:0/18:2)+NH4 | M+NH4 | TG(49:2) | TG |
| TG(15:1/16:0/18:2)+NH4 | M+NH4 | TG(49:3) | TG |
| TG(18:2/13:0/18:2)+NH4 | M+NH4 | TG(49:4) | TG |
| TG(18:0p/16:0/16:0)+NH4 | M+NH4 | TG(50:0p) | TG |
| TG(16:0e/16:0/18:1)+NH4 | M+NH4 | TG(50:1e) | TG |
| TG(16:0/16:0/18:2)+Na | M+Na | TG(50:2) | TG |
| TG(16:0/16:1/18:3)+H | M+H | TG(50:4) | TG |
| TG(16:0/16:1/18:3)+NH4 | M+NH4 | TG(50:4) | TG |
| TG(16:1/16:1/18:3)+H | M+H | TG(50:5) | TG |
| TG(16:1/16:1/18:3)+NH4 | M+NH4 | TG(50:5) | TG |
| TG(16:0/17:1/18:1)+NH4 | M+NH4 | TG(51:2) | TG |
| TG(15:0/18:1/18:2)+NH4 | M+NH4 | TG(51:3) | TG |
| TG(16:1/17:1/18:2)+NH4 | M+NH4 | TG(51:4) | TG |
| TG(15:1/18:2/18:2)+NH4 | M+NH4 | TG(51:5) | TG |
| TG(16:0/13:0/22:6)+NH4 | M+NH4 | TG(51:6) | TG |
| TG(18:0p/16:0/18:0)+NH4 | M+NH4 | TG(52:0p) | TG |
| TG(18:0e/16:0/18:1)+NH4 | M+NH4 | TG(52:1e) | TG |
| TG(18:0p/16:0/18:1)+H | M+H | TG(52:1p) | TG |
| TG(16:0/18:2/18:3)+NH4 | M+NH4 | TG(52:5) | TG |
| TG(16:1/18:2/18:3)+H | M+H | TG(52:6) | TG |
| TG(16:0/14:0/22:6)+NH4 | M+NH4 | TG(52:6) | TG |
| TG(18:2/17:1/18:2)+NH4 | M+NH4 | TG(53:5) | TG |
| TG(18:3/17:1/18:2)+NH4 | M+NH4 | TG(53:6) | TG |
| TG(18:0p/16:0/20:0)+NH4 | M+NH4 | TG(54:0p) | TG |
| TG(18:0/18:0/18:1)+NH4 | M+NH4 | TG(54:1) | TG |
| TG(20:0e/16:0/18:1)+NH4 | M+NH4 | TG(54:1e) | TG |
| TG(20:0p/16:0/18:1)+NH4 | M+NH4 | TG(54:1p) | TG |
| TG(16:0e/18:1/20:1)+NH4 | M+NH4 | TG(54:2e) | TG |
| TG(18:0p/18:1/18:2)+NH4 | M+NH4 | TG(54:3p) | TG |
| TG(18:1/18:1/18:2)+H | M+H | TG(54:4) | TG |
| TG(18:3/18:2/18:2)+H | M+H | TG(54:7) | TG |
| TG(18:3/18:2/18:2)+NH4 | M+NH4 | TG(54:7) | TG |
| TG(18:4/18:2/18:2)+NH4 | M+NH4 | TG(54:8) | TG |
| TG(19:1/18:2/18:2)+NH4 | M+NH4 | TG(55:5) | TG |
| TG(16:0/16:0/24:1)+NH4 | M+NH4 | TG(56:1) | TG |
| TG(20:0/18:1/18:1)+NH4 | M+NH4 | TG(56:2) | TG |
| TG(20:0e/18:1/18:1)+NH4 | M+NH4 | TG(56:2e) | TG |
| TG(18:0p/18:1/20:1)+NH4 | M+NH4 | TG(56:2p) | TG |
| TG(20:1/18:1/18:1)+NH4 | M+NH4 | TG(56:3) | TG |
| TG(20:0e/18:1/18:2)+NH4 | M+NH4 | TG(56:3e) | TG |
| TG(18:0/18:0/20:4)+NH4 | M+NH4 | TG(56:4) | TG |
| TG(18:1/18:1/20:3)+NH4 | M+NH4 | TG(56:5) | TG |
| TG(57:10)+NH4 | M+NH4 | TG(57:10) | TG |
| TG(18:2/18:2/21:1)+NH4 | M+NH4 | TG(57:5) | TG |
| TG(57:6)+NH4 | M+NH4 | TG(57:6) | TG |
| TG(16:0/18:1/24:0)+NH4 | M+NH4 | TG(58:1) | TG |
| TG(16:0/18:1/24:1)+NH4 | M+NH4 | TG(58:2) | TG |
| TG(18:1/18:2/22:0)+NH4 | M+NH4 | TG(58:3) | TG |
| TG(22:0/18:2/18:2)+NH4 | M+NH4 | TG(58:4) | TG |
| TG(26:0/16:0/18:1)+NH4 | M+NH4 | TG(60:1) | TG |
| TG(18:1/18:1/24:0)+NH4 | M+NH4 | TG(60:2) | TG |
| TG(18:1/18:1/24:1)+NH4 | M+NH4 | TG(60:3) | TG |
| TG(24:0/18:2/18:2)+NH4 | M+NH4 | TG(60:4) | TG |
| TG(24:1/18:2/18:2)+NH4 | M+NH4 | TG(60:5) | TG |
| TG(25:0/18:1/18:2)+NH4 | M+NH4 | TG(61:3) | TG |
| TG(25:1/18:1/18:2)+NH4 | M+NH4 | TG(61:4) | TG |
| TG(26:0/18:0/18:1)+NH4 | M+NH4 | TG(62:1) | TG |
| TG(26:0/18:1/18:1)+NH4 | M+NH4 | TG(62:2) | TG |
| TG(26:1/18:1/18:1)+NH4 | M+NH4 | TG(62:3) | TG |
| TG(26:0/18:2/18:2)+NH4 | M+NH4 | TG(62:4) | TG |
| TG(28:0/18:1/18:2)+NH4 | M+NH4 | TG(64:3) | TG |
| DG(16:0/16:1)+NH4 | M+NH4 | DG(32:1) | DG |
| DG(14:0/18:2)+NH4 | M+NH4 | DG(32:2) | DG |
| DG(16:0/18:1)+H | M+H | DG(34:1) | DG |
| DG(16:0/18:1)+NH4 | M+NH4 | DG(34:1) | DG |
| DG(16:0/18:2)+H | M+H | DG(34:2) | DG |
| DG(16:0/18:2)+NH4 | M+NH4 | DG(34:2) | DG |
| DG(16:0/18:3)+H | M+H | DG(34:3) | DG |
| DG(16:0/18:3)+NH4 | M+NH4 | DG(34:3) | DG |
| DG(34:3p)+H | M+H | DG(34:3p) | DG |
| DG(34:4)+H | M+H | DG(34:4) | DG |
| DG(18:0/18:1)+NH4 | M+NH4 | DG(36:1) | DG |
| DG(18:2/18:2)+NH4 | M+NH4 | DG(36:4) | DG |
| DG(18:3/18:2)+NH4 | M+NH4 | DG(36:5) | DG |
| DG(36:6e)+H | M+H | DG(36:6e) | DG |
| DG(20:0/18:1)+NH4 | M+NH4 | DG(38:1) | DG |
| DG(20:0/18:2)+NH4 | M+NH4 | DG(38:2) | DG |
| DG(20:5/18:2)+NH4 | M+NH4 | DG(38:7) | DG |
| DG(40:6p)+H | M+H | DG(40:6p) | DG |
| DG(22:5/18:2)+NH4 | M+NH4 | DG(40:7) | DG |
| DG(20:1/22:6)+NH4 | M+NH4 | DG(42:7) | DG |
| TG(36:3)+NH4 | M+NH4 | TG(36:3) | TG |
| TG(12:0/12:0/14:0)+NH4 | M+NH4 | TG(38:0) | TG |
| TG(10:0/12:0/18:1)+NH4 | M+NH4 | TG(40:1) | TG |
| TG(6:0/18:2/18:2)+NH4 | M+NH4 | TG(42:4) | TG |
| TG(16:0/9:0/18:2)+NH4 | M+NH4 | TG(43:2) | TG |
| TG(16:0/14:1/16:0)+NH4 | M+NH4 | TG(46:1) | TG |
| TG(16:0/8:0/22:6)+NH4 | M+NH4 | TG(46:6) | TG |
| TG(18:0p/14:0/16:0)+NH4 | M+NH4 | TG(48:0p) | TG |
| TG(16:0e/16:0/16:1)+NH4 | M+NH4 | TG(48:1e) | TG |
| TG(18:1p/14:0/16:0)+NH4 | M+NH4 | TG(48:1p) | TG |
| TG(16:0/16:0/16:2)+NH4 | M+NH4 | TG(48:2) | TG |
| TG(14:0e/16:0/18:2)+NH4 | M+NH4 | TG(48:2e) | TG |
| TG(12:0/18:2/18:2)+H | M+H | TG(48:4) | TG |
| TG(16:2/16:2/16:2)+NH4 | M+NH4 | TG(48:6) | TG |
| TG(16:1/10:0/22:6)+NH4 | M+NH4 | TG(48:7) | TG |
| TG(16:0/16:0/17:0)+NH4 | M+NH4 | TG(49:0) | TG |
| TG(16:0e/16:0/18:0)+NH4 | M+NH4 | TG(50:0e) | TG |
| TG(16:0/16:0/18:1)+H | M+H | TG(50:1) | TG |
| TG(16:0e/16:0/18:1)+H | M+H | TG(50:1e) | TG |
| TG(18:0p/16:0/16:1)+NH4 | M+NH4 | TG(50:1p) | TG |
| TG(16:0e/16:0/18:2)+H | M+H | TG(50:2e) | TG |
| TG(16:0e/16:0/18:2)+NH4 | M+NH4 | TG(50:2e) | TG |
| TG(16:0p/16:0/18:2)+NH4 | M+NH4 | TG(50:2p) | TG |
| TG(16:0/16:0/18:3)+H | M+H | TG(50:3) | TG |
| TG(16:0/16:0/18:3)+NH4 | M+NH4 | TG(50:3) | TG |
| TG(16:0e/16:1/18:2)+NH4 | M+NH4 | TG(50:3e) | TG |
| TG(16:0e/12:0/22:6)+NH4 | M+NH4 | TG(50:6e) | TG |
| TG(16:1/12:0/22:6)+NH4 | M+NH4 | TG(50:7) | TG |
| TG(16:0/17:0/18:1)+NH4 | M+NH4 | TG(51:1) | TG |
| TG(18:0p/16:0/18:0)+H | M+H | TG(52:0p) | TG |
| TG(18:0/16:0/18:1)+NH4 | M+NH4 | TG(52:1) | TG |
| TG(18:0e/16:0/18:1)+H | M+H | TG(52:1e) | TG |
| TG(18:0p/16:0/18:1)+NH4 | M+NH4 | TG(52:1p) | TG |
| TG(18:0e/16:0/18:2)+NH4 | M+NH4 | TG(52:2e) | TG |
| TG(18:0p/16:0/18:2)+H | M+H | TG(52:2p) | TG |
| TG(18:0p/16:0/18:2)+NH4 | M+NH4 | TG(52:2p) | TG |
| TG(18:1p/16:0/18:2)+NH4 | M+NH4 | TG(52:3p) | TG |
| TG(16:0/18:2/18:3)+H | M+H | TG(52:5) | TG |
| TG(16:0e/14:0/22:6)+NH4 | M+NH4 | TG(52:6e) | TG |
| TG(16:0p/18:3/18:3)+NH4 | M+NH4 | TG(52:6p) | TG |
| TG(16:1/14:0/22:6)+NH4 | M+NH4 | TG(52:7) | TG |
| TG(14:0e/16:1/22:6)+NH4 | M+NH4 | TG(52:7e) | TG |
| TG(14:0e/16:2/22:6)+NH4 | M+NH4 | TG(52:8e) | TG |
| TG(12:0/18:3/22:6)+NH4 | M+NH4 | TG(52:9) | TG |
| TG(17:0/18:1/18:1)+NH4 | M+NH4 | TG(53:2) | TG |
| TG(18:1/17:1/18:1)+NH4 | M+NH4 | TG(53:3) | TG |
| TG(18:1/17:1/18:2)+NH4 | M+NH4 | TG(53:4) | TG |
| TG(15:0/16:2/22:6)+NH4 | M+NH4 | TG(53:8) | TG |
| TG(18:0e/18:1/18:2)+NH4 | M+NH4 | TG(54:3e) | TG |
| TG(16:0e/18:2/20:2)+Na | M+Na | TG(54:4e) | TG |
| TG(18:1p/18:1/18:2)+NH4 | M+NH4 | TG(54:4p) | TG |
| TG(18:1/18:2/18:2)+NH4 | M+NH4 | TG(54:5) | TG |
| TG(16:0e/16:0/22:5)+H | M+H | TG(54:5e) | TG |
| TG(18:2/18:2/18:2)+H | M+H | TG(54:6) | TG |
| TG(16:0e/16:1/22:5)+H | M+H | TG(54:6e) | TG |
| TG(16:0e/16:1/22:6)+NH4 | M+NH4 | TG(54:7e) | TG |
| TG(14:0e/18:2/22:6)+NH4 | M+NH4 | TG(54:8e) | TG |
| TG(16:2/18:2/20:5)+NH4 | M+NH4 | TG(54:9) | TG |
| TG(14:0e/18:3/22:6)+NH4 | M+NH4 | TG(54:9e) | TG |
| TG(16:0/18:1/21:0)+NH4 | M+NH4 | TG(55:1) | TG |
| TG(19:1/18:1/18:2)+NH4 | M+NH4 | TG(55:4) | TG |
| TG(15:0/18:2/22:6)+NH4 | M+NH4 | TG(55:8) | TG |
| TG(15:1/18:2/22:6)+NH4 | M+NH4 | TG(55:9) | TG |
| TG(16:0/16:0/24:0)+NH4 | M+NH4 | TG(56:0) | TG |
| TG(18:3/18:2/20:5)+NH4 | M+NH4 | TG(56:10) | TG |
| TG(18:0/16:0/22:6)+NH4 | M+NH4 | TG(56:6) | TG |
| TG(16:0e/18:1/22:5)+H | M+H | TG(56:6e) | TG |
| TG(16:0e/18:0/22:6)+NH4 | M+NH4 | TG(56:6e) | TG |
| TG(16:0/18:1/22:6)+NH4 | M+NH4 | TG(56:7) | TG |
| TG(16:0e/18:1/22:6)+NH4 | M+NH4 | TG(56:7e) | TG |
| TG(18:0p/16:1/22:6)+NH4 | M+NH4 | TG(56:7p) | TG |
| TG(18:2/18:2/20:4)+NH4 | M+NH4 | TG(56:8) | TG |
| TG(16:0e/18:2/22:6)+H | M+H | TG(56:8e) | TG |
| TG(16:0e/18:2/22:6)+NH4 | M+NH4 | TG(56:8e) | TG |
| TG(16:0e/18:3/22:6)+NH4 | M+NH4 | TG(56:9e) | TG |
| TG(18:1p/16:2/22:6)+NH4 | M+NH4 | TG(56:9p) | TG |
| TG(16:0/18:1/23:0)+NH4 | M+NH4 | TG(57:1) | TG |
| TG(57:11)+NH4 | M+NH4 | TG(57:11) | TG |
| TG(18:1/18:1/21:0)+NH4 | M+NH4 | TG(57:2) | TG |
| TG(18:1/18:1/21:1)+NH4 | M+NH4 | TG(57:3) | TG |
| TG(18:2/18:2/21:0)+NH4 | M+NH4 | TG(57:4) | TG |
| TG(18:2/18:2/22:6)+NH4 | M+NH4 | TG(58:10) | TG |
| TG(18:2p/18:2/22:6)+NH4 | M+NH4 | TG(58:10p) | TG |
| TG(18:3/18:2/22:6)+NH4 | M+NH4 | TG(58:11) | TG |
| TG(22:1/18:2/18:2)+NH4 | M+NH4 | TG(58:5) | TG |
| TG(18:1/18:1/22:4)+NH4 | M+NH4 | TG(58:6) | TG |
| TG(18:0/18:1/22:6)+NH4 | M+NH4 | TG(58:7) | TG |
| TG(18:0e/18:1/22:6)+NH4 | M+NH4 | TG(58:7e) | TG |
| TG(18:0p/18:1/22:6)+NH4 | M+NH4 | TG(58:7p) | TG |
| TG(18:0e/18:2/22:6)+NH4 | M+NH4 | TG(58:8e) | TG |
| TG(18:0p/18:2/22:6)+H | M+H | TG(58:8p) | TG |
| TG(18:0p/18:2/22:6)+NH4 | M+NH4 | TG(58:8p) | TG |
| TG(18:1p/18:2/22:6)+NH4 | M+NH4 | TG(58:9p) | TG |
